# Dynamic monocyte changes as prognostic indicators in operable gastric cancer: a retrospective cohort analysis

**DOI:** 10.3389/fonc.2025.1514281

**Published:** 2025-02-07

**Authors:** Yiwei Jiang, Xianwei Sun, Chen Yang, Dandan Song, Chongjun Zhou, Xinxin Chen, Chongquan Huang, Zhonglin Wang, Jiante Li

**Affiliations:** ^1^ Department of Gastrointestinal Surgery, The 2nd Affiliated Hospital and Yuying Children’s Hospital of Wenzhou Medical University, Wenzhou, Zhejiang, China; ^2^ Department of Gastrointestinal Surgery, The 1st Affiliated Hospital Wenzhou Medical University, Wenzhou, Zhejiang, China; ^3^ Department of Anorectal Surgery, The 2nd Affiliated Hospital and Yuying Children’s Hospital of Wenzhou Medical University, Wenzhou, Zhejiang, China; ^4^ Department of Radioimaging, Wenzhou Central Hospital, Wenzhou, Zhejiang, China

**Keywords:** gastric cancer, postoperative and preoperative monocytes ratio, prognosis, overall survival (OS), precise treatment

## Abstract

**Objective:**

This study aims to elucidate the relationship between postoperative monocyte count and gastric cancer prognosis. We introduce a standardized monocyte ratio (MMR) to predict postoperative survival rates in gastric cancer patients effectively.

**Methods:**

A test cohort was created to develop and evaluate the pre- and postoperative MMR as a mortality predictor in gastric cancer patients. We used Kaplan-Meier survival analysis, complemented by univariate and multivariate analyses. The predictive utility of MMR was assessed via time-dependent ROC curves and decision-curve analysis.

**Results:**

The sample distributions in both cohorts were similar. The MMR showed high predictive value and significant clinical benefits in 1, 3, and 5-year overall survival (OS) assessments. These findings enhance understanding of prognosis and aid in developing more precise treatment plans.

**Conclusions:**

MMR is confirmed as an independent factor in predicting overall survival in gastric cancer patients, proving to be a reliable and cost-effective prognostic indicator.

## Introduction

Currently, gastric cancer is recognized as the fifth most common malignancy globally and is the fourth leading cause of cancer-related deaths (1). Systemic therapies for gastric cancer, including neoadjuvant chemotherapy, radiotherapy, and combination therapies. Pathological findings are the cornerstone for cancer classification, prediction, and research. However, even with the use of the TNM staging system, patients at the same disease stage often experience varying clinical outcomes (2). This discrepancy highlights the critical need for additional markers to accurately predict individual clinical outcomes (3, 4). Identifying such markers promises to significantly improve clinical benefits for our patients.

Monocytes, a type of immune cell derived from the myeloid lineage, have been extensively studied due to their strong association with cancer cell growth, invasiveness, and metastasis (5–7). Upon recruitment to the tumor microenvironment (TME), monocytes differentiate into macrophages, which exhibit diverse functional properties. These macrophages can be polarized into the M1 phenotype, which exhibits anti-tumor properties, or the M2 phenotype, which promotes tumor growth and immune suppression. The balance between these macrophage subtypes within the TME has profound implications for tumor progression, metastasis, and the response to therapy. M1 macrophages generally exert anti-cancer effects by stimulating pro-inflammatory responses and activating cytotoxic immune cells, while M2 macrophages contribute to tumor growth, angiogenesis, and immune evasion through the secretion of anti-inflammatory cytokines like IL-10 and TGF-β (8–10).

Recent studies have emphasized the prognostic value of preoperative monocyte counts in predicting survival outcomes in cancer patients (6, 11). However, factors like gender, age, and regional differences add complexity to using preoperative monocyte count.Therefore, it is crucial to evaluate the relevance of comparing pre- and postoperative monocyte counts. Analyzing the resulting ratio of postoperative to preoperative monocyte counts could be a valuable measure for clinical use and personalized prognosis in gastric cancer patients.

Numerous parameters involving peripheral blood cell analysis have been introduced to assess survival outcomes in cancer patients (12–14). These parameters often focus on measuring inflammation, which is known to be linked to cancer development and progression. They typically involve calculating ratios of different blood cells related to neutrophils, lymphocytes, and monocytes, such as the neutrophil/lymphocyte ratio and the monocyte/lymphocyte ratio (15–17) and the monocyte/lymphocyte ratio (18–20). It’s important to acknowledge that tumor development and progression are dynamic processes, and the predictive accuracy of certain preoperative indicators for postoperative survival may not be fully reliable. Furthermore, standardizing these indicators is challenging due to variations in detection methods and equipment. Considering these limitations, we have introduced the MMR and established a test cohort. Our research indicates that MMR independently correlates with overall survival in gastric cancer patients, thus confirming its value as a precise prognostic tool.

## Method

### Clinicopathological data

**Figure 1:**
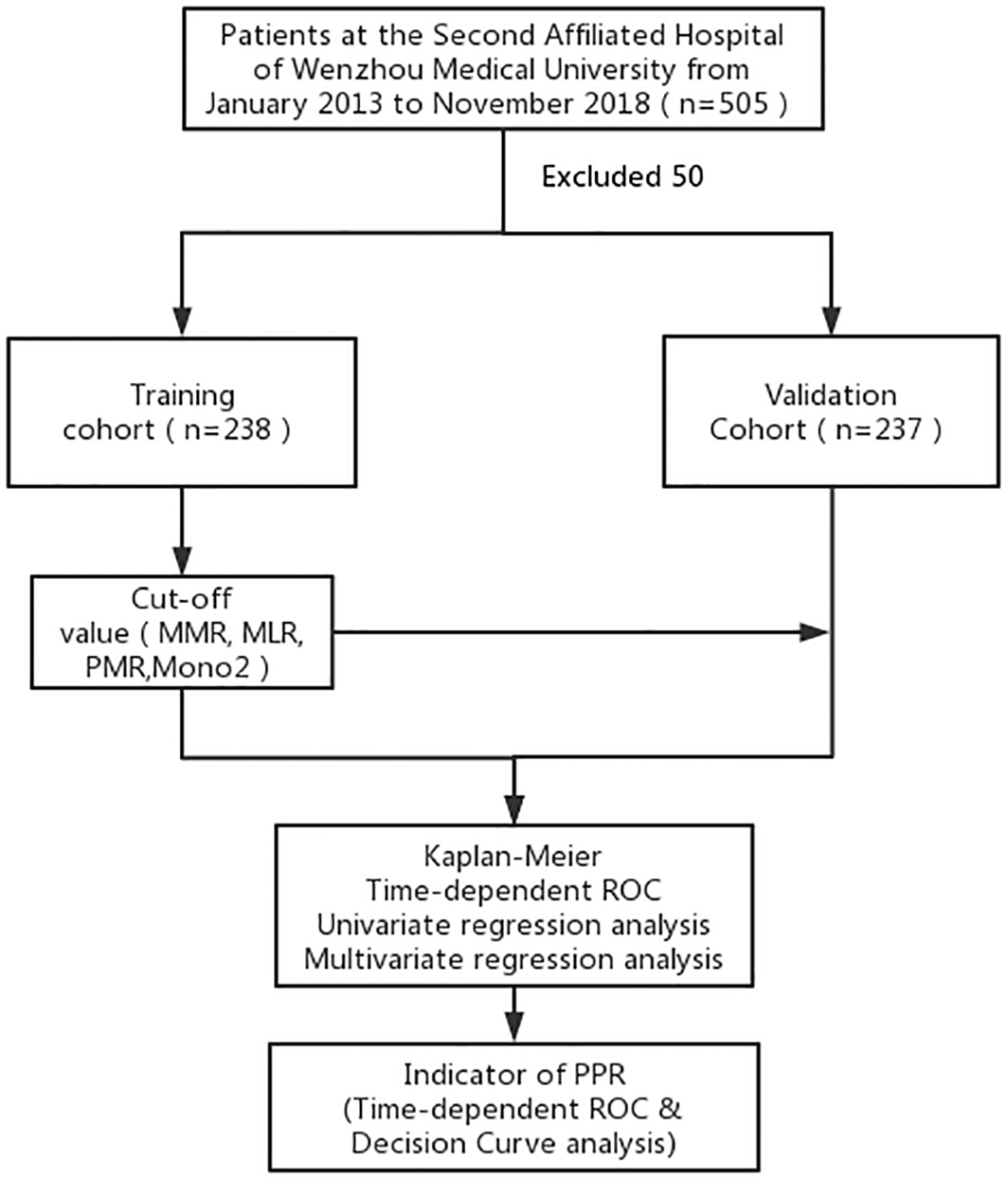
A standard flow chart of the patients in this study. MMR, the postoperative monocyte/preoperative monocyte ratio.

Our study involved a patient cohort treated from 2014 to 2018. Each participant underwent a radical gastrectomy, as depicted in **Figure 1**, and was selected based on detailed histopathological examinations. We established strict exclusion criteria: a history of any malignancy, use of anti-monocyte drugs, monocyte-related diseases, stage IV cancer, previous neoadjuvant chemotherapy, or loss to follow-up. We meticulously collected essential clinical data from medical records, including demographic information (age, gender), clinical data (TNM staging, tumor location and size, histological grade, neurovascular infiltration), and laboratory data (MMR, monocyte/lymphocyte ratio (MLR), platelet monocyte ratio (PMR)). Additionally, we gathered patient survival information via telephone follow-ups.

### Laboratory parameters

To mitigate the impact of surgical stress on monocyte counts, blood samples were collected at least two weeks after surgery. This timing was chosen to establish baseline values within the week before surgery, ensuring an accurate representation of the preoperative status. Additionally, to account for the hidden effects of postoperative chemotherapy on bone marrow function, complete blood counts were performed before starting postoperative chemotherapy. For determining the most effective cut-off points, we employed the X-tile software (21).Within the Development cohort, the optimal cut-off values were identified as MMR (2.03), MLR (0.21), and PMR (530). These specific thresholds are crucial in understanding the changes in monocyte counts during the postoperative period. They offer critical insights into the complex interactions between surgical stress, chemotherapy, and blood parameters.

### Statistical analysis

In the statistical analysis, SPSS was employed. We presented descriptive statistics for continuous variables with non-normal distributions using the median and interquartile range. Categorical variables were displayed in terms of frequency and proportion, and we utilized the χ^2^ test for their statistical analysis. The Kaplan–Meier method was applied to assess overall survival (OS). The performance of the predictive model was evaluated using time-dependent ROC curves. We examined the relationship between risk factors through both univariate and multivariate logistic regression analyses. The multivariate regression analysis included only those variables that showed statistically significant differences in the univariate survival analysis. Additionally, we constructed a logistic regression model to establish a joint index model. The net benefit of this model, represented as MMR, was estimated using decision curves. A p-value below 0.05 was considered statistically significant.

## Result

### Clinical features

The clinical characteristics and clinicopathological data of the patients are detailed in **Table 1**. Both cohorts display similar distributions of key baseline variables in their clinical data. The survival curves for all patients across the two cohorts are illustrated in **Supplementary Figure S1**, indicating no statistically significant differences in overall survival (OS) probabilities.

**Table 1:** Clinicopathological data and clinical characteristics of patients in the training and validation cohorts.

Characteristics	Development Cohort	Teat cohort	P value
N	238	237	
Age, median (IQR)	63.5 (56, 73)	63 (56, 73)	0.938
gender, n (%)			0.271
Male	167 (35.2%)	177 (37.3%)	
Female	71 (14.9%)	60 (12.6%)	
T.stage, n (%)			0.751
T1-T2	99 (20.8%)	102 (21.5%)	
T3-T4	139 (29.3%)	135 (28.4%)	
N.stage, n (%)			0.552
No	105 (22.1%)	111 (23.4%)	
Yes	133 (28%)	126 (26.5%)	
Pathologic.stage, n (%)			0.723
Stage I	85 (17.9%)	82 (17.3%)	
Stage II	48 (10.1%)	55 (11.6%)	
Stage III	105 (22.1%)	100 (21.1%)	
Tumor size, n (%)			0.480
<5cm	158 (33.3%)	150 (31.6%)	
≥5cm	80 (16.8%)	87 (18.3%)	
Microvascular invasion, n (%)			0.822
No	138 (29.1%)	135 (28.4%)	
Yes	100 (21.1%)	102 (21.5%)	
MMR, median (IQR)	1.932 (1.5148, 2.6179)	2.1375 (1.5758, 2.9026)	0.065
MLR, median (IQR)	0.25733 (0.19962, 0.31957)	0.25914 (0.18099, 0.32056)	0.700
PMR, median (IQR)	496.35 (340.02, 790.91)	564.44 (426.32, 791.51)	0.070

AJCC, American Joint Committee on Cancer; MMR, postoperative/preoperative monocyte ratio; MLR, monocyte/lymphocyte ratio; PMR, platelet monocyte ratio.

### Survival analysis

**Figure 2:**
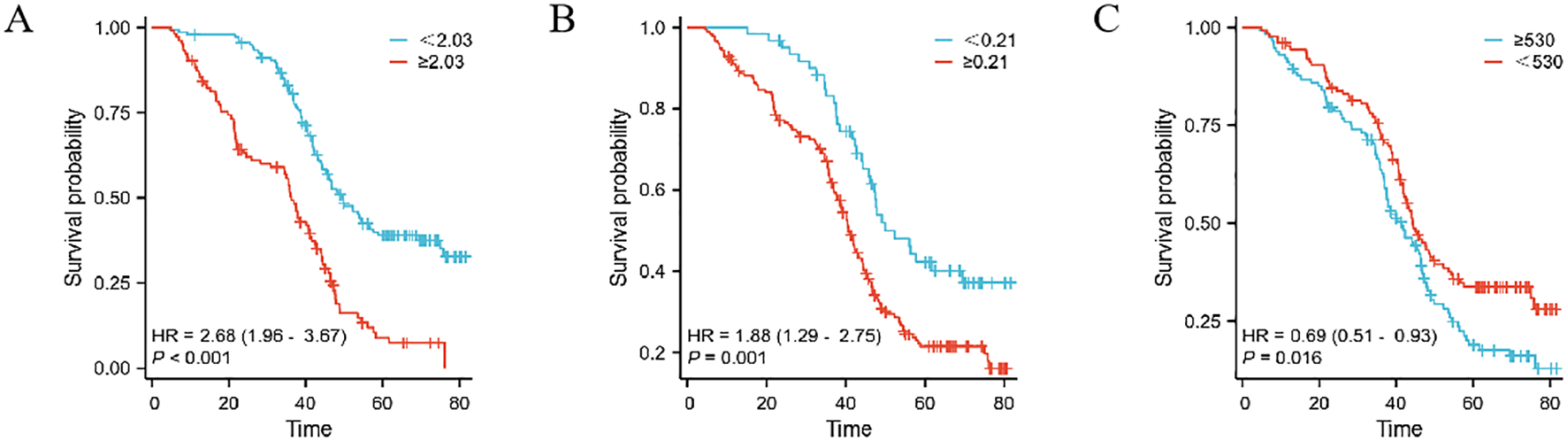
The prognostic significance in patients with gastric cancer in the Development cohort. The postoperative monocyte/preoperative monocyte ratio(MMR) **(A)**, monocyte/lymphocyte ratio (MLR) **(B)** and postoperative monocyte/preoperative monocyte ratio (PMR) **(C)**.

**Figure 3:**
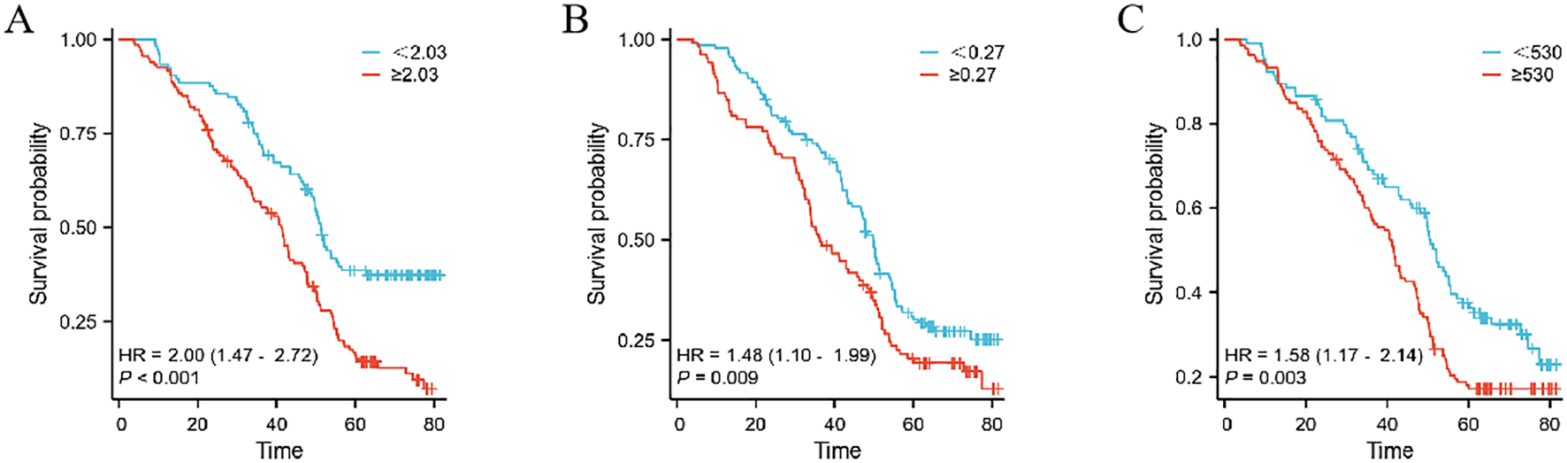
The prognostic significance in patients with gastric cancer in the Teat cohort.The postoperative monocyte/preoperative monocyte ratio (MMR) **(A)**, neutrophil/lymphocyte ratio (MLR) **(B)** and postoperative monocyte/preoperative monocyte ratio (PMR) **(C)**.

This study extensively examined the relationship between prognostic indicators - specifically, the MMR, Monocyte/Lymphocyte Ratio (MLR), and Platelet/Monocyte Ratio (PMR) - and their impact on patient outcomes. In the Development cohort, we observed that an elevated postoperative/preoperative MMR, alongside MLR and PMR, was strongly associated with reduced overall survival (OS) in gastric cancer patients undergoing radical resection. Specifically, the hazard ratios were as follows: MMR: 2.68 (1.96–3.67, p < 0.001), MLR: 1.88 (1.29–2.75, p = 0.001), and PMR: 0.69 (0.51–0.93, p = 0.016) (as shown in **Figure 2**). Similarly, in the Teat cohort, a significant link between MMR and decreased OS was found in patients undergoing the same surgical procedure, with hazard ratios of 2.00 (1.47–2.72, p < 0.001), 1.48 (1.10–1.99, p = 0.009), and 1.58 (1.17–2.14, p = 0.003) for MMR, MLR, and PMR, respectively (refer to **Figure 3**).

### Univariate and multivariate analyses

To evaluate prognostic factors in gastric cancer patients undergoing radical resection, we executed both univariate and multivariate analyses. The univariate analysis of the Development cohort pinpointed critical prognostic factors: age, monocyte-to-preoperative MMR, monocyte/lymphocyte ratio (MLR), and platelet/monocyte ratio (PMR), as detailed in **Table 2**. Furthermore, the multivariate analysis identified MMR and MLR as independent predictors of outcome. Within the test cohort, significant prognostic factors identified through univariate analysis included age, MMR, and PMR. Multivariate analysis reinforced the role of age, MMR, and PMR as independent predictors, as shown in **Table 3**. These findings underscore MMR’s role as a standalone predictor of overall survival in gastric cancer patients, offering a prognostic value that surpasses both MLR and PMR.

**Table 2:** Univariate and multivariate cox regression analyses for overall survival in patients with colorectal cancer of development cohort.

Characteristics	Univariate analysis		Multivariate analysis	
	Odds Ratio (95% CI)	P value	Odds Ratio (95% CI)	P value
Age	1.062 (1.035 - 1.090)	< 0.001*	1.055 (1.026 - 1.085)	< 0.001*
Gender				
Male Vs Female	0.620 (0.344 - 1.115)	0.110		
T.stage				
T1-T2 Vs T3-T4	1.053 (0.603 - 1.840)	0.856		
N.stage				
Yes Vs No	1.102 (0.632 - 1.922)	0.733		
Pathologic.stage				
Stage I Vs Stage II	1.122 (0.509 - 2.470)	0.775		
Stage I Vs Stage III	0.833 (0.449 - 1.547)	0.563		
Tumor size				
<5cm Vs ≥5cm	0.806 (0.453 - 1.435)	0.464		
Microvascular invasion				
Yes Vs No	0.947 (0.541 - 1.656)	0.848		
MMR	3.500 (1.878 - 6.524)	< 0.001*	3.630 (1.777 - 7.416)	< 0.001*
MLR	2.262 (1.233 - 4.148)	0.008*	3.185 (1.519 - 6.682)	0.002*
PMR	1.855 (1.054 - 3.263)	0.032*	1.677 (0.840 - 3.349)	0.143

CI, Confidence interval; MMR, postoperative/preoperative monocyte ratio; MLR, monocyte/lymphocyte ratio; PMR, platelet monocyte ratio.

*Statistically significant.

**Table 3:** Univariate and multivariate cox regression analyses for overall survival in patients with colorectal cancer of validate cohort.

Characteristics	Univariate analysis		Multivariate analysis	
	Odds Ratio (95% CI)	P value	Odds Ratio (95% CI)	P value
Age	0.973 (0.948 - 1.000)	0.047*	0.971 (0.943 - 1.000)	0.049*
Gender				
Male Vs Female	1.120 (0.563 - 2.227)	0.746		
T.stage				
T1-T2 Vs T3-T4	0.964 (0.532 - 1.749)	0.905		
N.stage				
Yes Vs No	1.394 (0.768 - 2.532)	0.275		
Pathologic.stage				
Stage I vs Stage II	1.452 (0.620 - 3.398)	0.391		
Stage I vs Stage III	0.790 (0.407 - 1.534)	0.486		
Tumor size				
<5cm Vs ≥5cm	1.301 (0.697 - 2.429)	0.408		
Microvascular invasion				
Yes Vs No	1.390 (0.770 - 2.512)	0.275		
MMR	4.158 (2.206 - 7.835)	< 0.001*	4.487 (2.216 - 9.086)	< 0.001*
MLR	1.782 (0.964 - 3.293)	0.065	3.930 (1.855 - 8.327)	< 0.001*
PMR	2.304 (1.264 - 4.200)	0.006*	2.253 (1.094 - 4.640)	0.028*

CI, Confidence interval; MMR, postoperative/preoperative monocyte ratio; MLR, monocyte/lymphocyte ratio; PMR, platelet monocyte ratio.

*Statistically significant.

### Predictive value for prognosis of different coefficients

**Figure 4:**
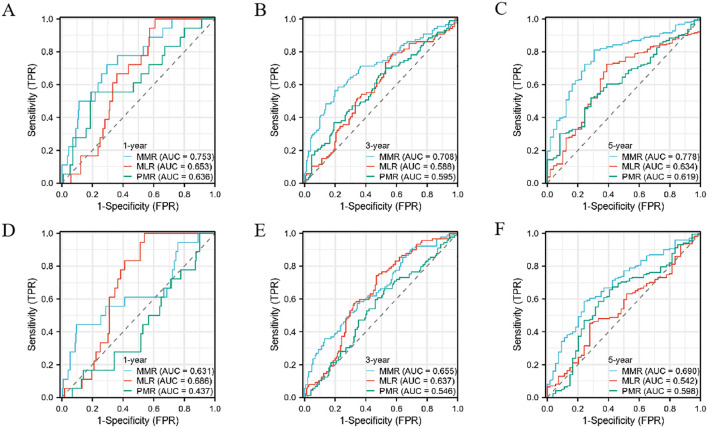
The predictive ability of the MMR, MLR, PMR, by time-dependent receiver operating characteristic (ROC) curves in 1 year, 3 years, and 5 years in the Development cohort **(A–C)** and Teat cohort **(D–F)** in gastric cancer.

In the Development cohort, composed of patients undergoing radical gastrectomy for gastric cancer, a time-dependent Receiver Operating Characteristic (ROC) analysis revealed the superior prognostic efficacy of the postoperative MMR, as indicated by its area under the curve (AUC) values. Specifically, MMR demonstrated AUC values of 0.753, 0.708, and 0.778 for 1-year, 3-year, and 5-year overall survival (OS), respectively. These figures indicate a more robust predictive performance compared to other indicators, as shown in **Figures 4A–C**. In the Teat cohort, MMR also displayed enhanced predictive abilities relative to both the monocyte lymphocyte ratio (MLR) and the platelet monocyte ratio (PMR), evidenced by higher AUC values, detailed in **Figures 4D–F**.

### Prognostic value of MMR

**Figure 5:**
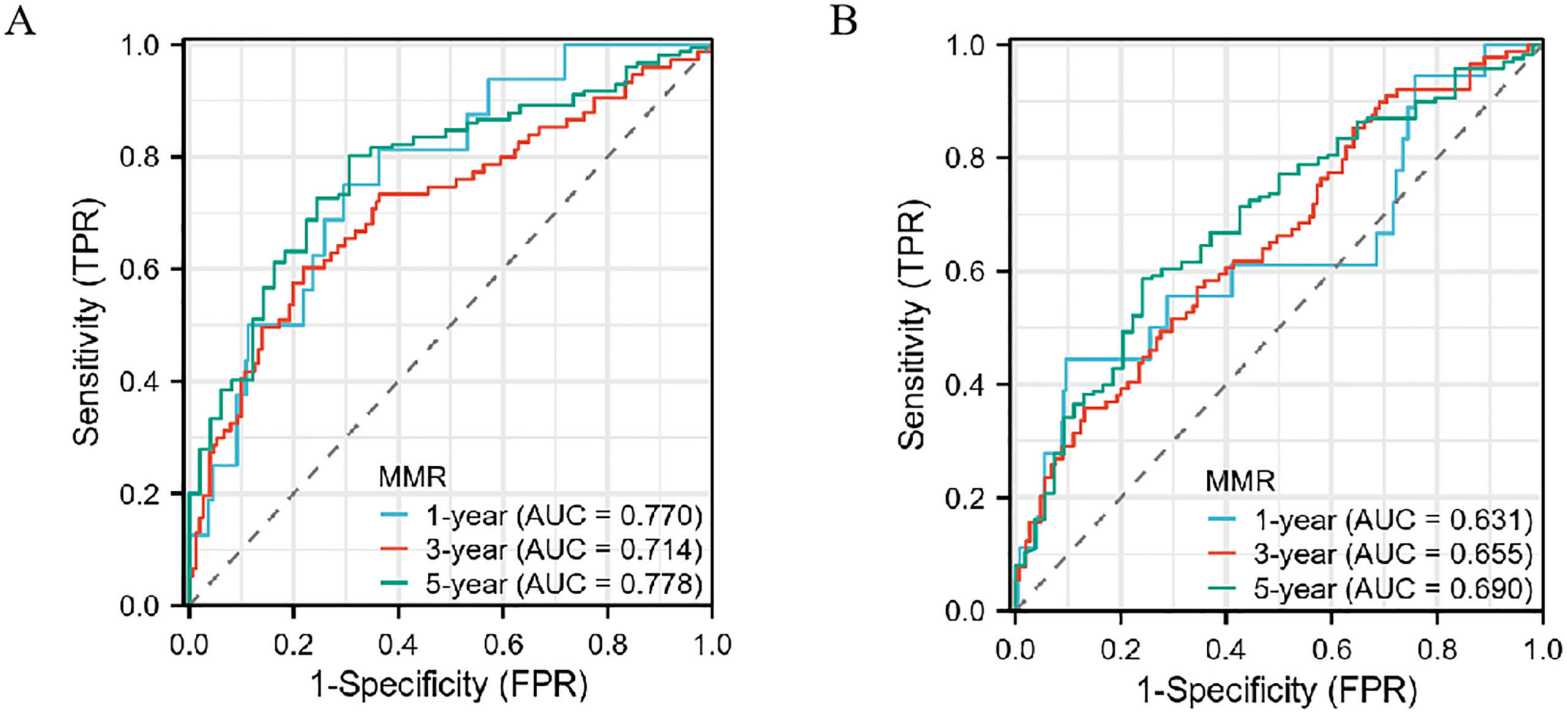
The predictive ability of the MMR in gastric cancer by time-dependent receiver operating characteristic (ROC) curves in the Development cohort **(A)** and Teat cohort **(B)** in 1 year, 3 years, and 5 years.

Decision curve analysis indicates that the postoperative/preoperative MMR offers the highest net benefit in predicting outcomes for gastric cancer within the Development cohort (see **Supplementary Figures S2A–C**). This superior net benefit is also observed in the Teat cohort, further establishing MMR as a robust prognostic indicator (refer to **Supplementary Figures S2D–F**). These results highlight MMR’s reliability in prognostic evaluation. Given its impressive performance, an in-depth investigation into the impact of MMR on cancer prognosis is essential. Notably, the 5-year overall survival rate reaches its peak in both the Development and Teat cohorts, with the 1-year and 3-year overall survival rates showing similar trends across these groups (as illustrated in **Figures 5A, B**).

## Discussion

In recent years, considerable research has focused on deciphering the complex processes behind monocytes’ role in tumor development and metastasis. Influenced by tumor cells’ regulatory mechanisms, monocytes demonstrate the ability to migrate into various tissues (22). Similarly, monocytes can be recruited to the TME. Influenced by local factors in this environment (23, 24), they undergo differentiation and subsequently release cytokines and elements that contribute to tumor progression (25, 26). Modifying the transcriptional programs of these tumor-infiltrating, monocyte-derived cells has shown promising anti-tumor effects in preclinical models (27, 28). A variety of strategies have been developed to directly modulate these cells. These include interventions that target key signaling pathways linked to immune suppression, such as STAT3, NF-κB, and PI3Kγ pathways. Furthermore, synergistic approaches are showing promise in enhancing pharmacological activities. For example, targeting the PI3Kγ pathway not only hinders the growth of tumor vasculature but also reduces local immunosuppression. This is achieved by decreasing the number of immunosuppressive Tumor-Associated Macrophages (TAMs) and encouraging Tumor-Associated Dendritic Cells (TADCs) to produce cytokines. These cytokines, in turn, stimulate effective T cell responses, contributing to a more robust immune response against the tumor (29). The CD40:CD40 ligand axis presents itself as an appealing target for transforming immunosuppressive cells into effective Antigen-Presenting Cells (APCs). Therapies that utilize agonistic CD40 antibodies have demonstrated effectiveness in disrupting the communication between Myeloid-Derived Suppressor Cells (MDSCs) and Regulatory T cells (Tregs). These therapies not only convert MDSCs into functional cells that prime T cells but also induce tumoricidal activity in Tumor-Associated Macrophages (TAMs) (29). Enhancing our comprehension of the mechanisms that govern monocyte differentiation within the Tumor Microenvironment (TME) is crucial for developing future therapies. It is particularly important to decode the complex behaviors of tumor-infiltrating myeloid cells, utilizing cutting-edge technologies like single-cell RNA sequencing. This precise mapping will facilitate a thorough understanding of the processes by which monocytes evolve into cells that either promote or inhibit tumor growth.

The prognostic evaluation of preoperative monocyte count in patients with solid tumors has undergone extensive investigation. Nevertheless, certain issues warrant additional exploration (30–32). Firstly, the clinical utility of preoperative monocyte counts faces challenges due to a lack of standardization and individualization. This issue stems from variations across individuals, instruments, and geographical locations. Secondly, the link between increased postoperative monocyte count and the risk of cancer recurrence, along with its impact on prognosis, is not yet fully understood. To address these challenges, we have introduced the MMR, which is derived from both post- and pre-operative monocyte counts. This method is designed to normalize variations in baseline values that arise from personal, regional, and instrumental differences. As a result, the MMR effectively captures an individual’s monocyte dynamics, making it a more reliable and standardized indicator. This enhances its value in assessing the relationship between postoperative monocyte counts and survival outcomes in patients undergoing radical resection for gastric cancer. However, it is important to acknowledge the limitations of the MMR in fully capturing the complexity of the TME and the role of TAMs. Monocyte polarization is a highly intricate process that generates a variety of macrophage phenotypes, extending far beyond the traditional M1 and M2 classifications. These diverse macrophage subsets contribute to a range of immune responses, from pro-inflammatory activity (M1) to immune suppression (M2), and their interactions with tumor cells and other infiltrating immune cells are pivotal in determining tumor progression, metastasis, and response to therapy. The MMR, while offering a standardized measure of overall monocyte dynamics, does not directly account for the full spectrum of macrophage polarization and its interactions within the tumor microenvironment. Thus, while MMR provides an important prognostic tool, it should be interpreted with caution, as it may not fully reflect the intricate and dynamic interactions between different macrophage subsets, tumor cells, and other immune infiltrates. Further research is needed to explore the relationship between monocyte polarization, TAM phenotypes, and clinical outcomes in gastric cancer.

This study investigated the prognostic assessment capabilities of three factors—the MLR, PMR, and MMR—in patients diagnosed with gastric cancer at two independent medical centers. Our analysis identified a significant correlation between elevated MMR levels and unfavorable prognosis in patients undergoing radical resection for gastric cancer. Notably, MMR stood out as an independent predictive factor for overall survival in both the primary and Teat cohorts. In comparison to NLR and MLR, MMR displayed a superior prognostic value in predicting patient outcomes across both cohorts. These findings suggest that a sustained increase in postoperative monocyte levels is associated with negative patient outcomes, implying that a rise in monocyte count may contribute to tumor cell resurgence and proliferation after surgical intervention. Therefore, dynamic monitoring of postoperative monocyte levels is promising as an indicative measure for patient prognosis. Interestingly, upon reviewing the data, we found that while staging traditionally plays a critical role in determining prognosis, it was not associated with prognosis in our cohort. We speculate that although staging remains an important prognostic factor, its predictive power may be moderated by other clinical variables, such as the use of neoadjuvant therapy and the overall tumor burden at diagnosis.

The association between the MMR and prognosis in gastric cancer may be attributed to several potential mechanisms that reflect the complex interplay between the immune system and TME. The MMR in gastric cancer reflects the balance between two macrophage subtypes, M1 and M2, which play opposite roles in tumor progression. M1 macrophages promote anti-tumor immunity by releasing pro-inflammatory cytokines and activating immune cells like cytotoxic T-cells, while M2 macrophages support tumor growth and metastasis by fostering immune suppression, angiogenesis, and extracellular matrix remodeling. A higher MMR, with more monocytes than macrophages, suggests an inflammatory environment that favors M1 polarization and a better prognosis. Conversely, a lower MMR, with more M2 macrophages, indicates immune evasion and worse clinical outcomes. The MMR also reflects the systemic inflammatory state, influencing tumor progression and response to treatments like immunotherapy and chemotherapy. Therefore, MMR serves as a potential prognostic marker for gastric cancer by indicating immune dynamics in the tumor microenvironment.

Given the significant predictive efficacy of the postoperative/preoperative MMR, we conducted an extensive analysis to determine its prognostic significance in patients undergoing radical resection for gastric cancer. Crucially, the inclusion of MMR indicators revealed enhanced predictive capabilities, consistently demonstrating a strong prognostic ability in both the experimental and test groups.

The evaluation of peripheral blood counts plays a vital role in preoperative assessments and postoperative follow-up protocols. In this context, MMR emerges as an accessible, cost-effective, and reliably reproducible parameter. However, it’s important to acknowledge certain limitations of our study. Firstly, the preoperative and postoperative peripheral blood counts were collected from the same institution. A single-center study may limit the generalizability of the findings. Our findings should be validated in a more diverse and broader cohort to confirm the robustness and applicability of the MMR as a prognostic tool in gastric cancer. Secondly, our study was based on a retrospective cohort design, which can introduce biases and limit the ability to establish causal relationships. Some critical factors, such as treatment methods, postoperative complications, nutritional status, severe preoperative infections, significant organic lesions in vital organs, or autoimmune diseases, were not available in the data collection process. Future prospective studies with more comprehensive data collection could minimize potential confounders and enhance the accuracy of the prognostic model. Thirdly, our analysis did not consider progression-free survival as an outcome measure. Lastly, the exclusive focus on OS is a limitation and that future research should consider both OS and progression-free survival.

## Conclusion

MMR serves as a dependable metric for evaluating survival outcomes following radical resection in gastric cancer patients. An elevated MMR is strongly associated with a negative prognosis, providing a more objective standard for predicting survival after such procedures. Additionally, MMR facilitates the stratification of mortality risk, enabling healthcare professionals to develop more personalized and effective treatment strategies.

## Data Availability

The raw data supporting the conclusions of this article will be made available by the authors, without undue reservation.

## References

[1] Michael Brian LaPelusa CS Gibson MK. Demographic differences in the treatment of gastric cancer. Gastrointestinal Cancer. (2022). doi: 10.1200/JCO.2022.40.16_suppl.e16078

[2] Lin J-X Desiderio J Lin J-P Wang W Tu R-H Li P. Multicenter validation study of the american joint commission on cancer (8th edition) for gastric cancer: proposal for a simplified and improved TNM staging system. J Cancer. (2020) 11:3483–91. doi: 10.7150/jca.36891 PMC7150461 32284744

[3] Bando E Makuuchi R Irino T Tanizawa Y Kawamura T Terashima M. Validation of the prognostic impact of the new tumor-node-metastasis clinical staging in patients with gastric cancer. Gastric Cancer. (2018) 22:123–9. doi: 10.1007/s10120-018-0799-9 29357013

[4] Cao H Tang Z Yu Z Wang Q Li Z Lu Q. Comparison of the 8th union for international cancer control lymph node staging system for gastric cancer with two other lymph node staging systems. Oncol Lett. (2018). doi: 10.3892/ol.2018.9694 PMC6312982 30655898

[5] Feng AL Zhu JK Sun JT Yang MX Neckenig MR Wang XW. CD16+ monocytes in breast cancer patients: expanded by monocyte chemoattractant protein-1 and may be useful for early diagnosis. Clin Exp Immunol. (2011) 164:57–65. doi: 10.1111/j.1365-2249.2011.04321.x 21361908 PMC3074217

[6] Shibutani M Maeda K Nagahara H Fukuoka T Nakao S Matsutani S. The peripheral monocyte count is associated with the density of tumor-associated macrophages in the tumor microenvironment of colorectal cancer: a retrospective study. BMC Cancer. (2017) 17. doi: 10.1186/s12885-017-3395-1 PMC5460583 28583114

[7] Liu T Larionova I Litviakov N Riabov V Zavyalova M Tsyganov M. Tumor-associated macrophages in human breast cancer produce new monocyte attracting and pro-angiogenic factor YKL-39 indicative for increased metastasis after neoadjuvant chemotherapy. OncoImmunology. (2018) 7. doi: 10.1080/2162402X.2018.1436922 PMC5980380 29872578

[8] Burgess B Levine B Taylor RN Kelly MG. Preoperative circulating lymphocyte and monocyte counts correlate with patient outcomes in type I and type II endometrial cancer. Reprod Sci. (2020) 27:194–203. doi: 10.1007/s43032-019-00009-4 32046381 PMC11758426

[9] Shibutani M Maeda K Nagahara H Iseki Y Ikeya T Hirakawa K. Prognostic significance of the preoperative lymphocyte-to-monocyte ratio in patients with colorectal cancer. Oncol Lett. (2017) 13:1000–6. doi: 10.3892/ol.2016.5487 PMC5351160 28356991

[10] Haruki K Shiba H Fujiwara Y Furukawa K Iida T Ohkuma M. Preoperative peripheral blood neutrophil count predicts long-term outcomes following hepatic resection for colorectal liver metastases. Oncol Lett. (2017) 13:3688–94. doi: 10.3892/ol.2017.5873 PMC5431379 28521471

[11] Skubitz KM Domingo-Musibay E Lindgren BR Cheng EY. Prospective trial of neutrophil/lymphocyte ratio and other blood counts as biomarkers of survival among patients with high-grade soft tissue sarcomas treated with pegylated liposomal doxorubicin and ifosfamide. Cancers. (2022) 14. doi: 10.3390/cancers14143419 PMC9316699 35884480

[12] Diaz C Calderillo-Ruiz G Ramos-Ramirez M Herrera M Manuel F Horacio L. Association of Prognostic Nutritional Index as a predictive factor of survival in patients with colorectal cancer in a Mexican population. Ann Oncol. (2019) 30. doi: 10.1093/annonc/mdz155.342

[13] Fukuhara M Muto S Inomata S Yamaguchi H Mine H Takagi H. The clinical significance of tertiary lymphoid structure and its relationship with peripheral blood characteristics in patients with surgically resected non-small cell lung cancer: a single-center, retrospective study. Cancer Immunol Immunother. (2021) 71:1129–37. doi: 10.1007/s00262-021-03067-3 PMC10992741 34596720

[14] Onagi H Horimoto Y Sakaguchi A Ikarashi D Yanagisawa N Nakayama T. High platelet-to-lymphocyte ratios in triple-negative breast cancer associates with immunosuppressive status of TILs. Breast Cancer Res. (2022) 24. doi: 10.1186/s13058-022-01563-7 PMC9552414 36217150

[15] Belkouchi Y Nebot-Bral L Lawrance L Kind M David C Ammari S. Predicting immunotherapy outcomes in patients with MSI tumors using NLR and CT global tumor volume. Front Oncol. (2022) 12. doi: 10.3389/fonc.2022.982790 PMC9641225 36387101

[16] Gambardella C Mongardini FM Paolicelli M Bentivoglio D Cozzolino G Ruggiero R. Role of inflammatory biomarkers (NLR, LMR, PLR) in the prognostication of Malignancy in indeterminate thyroid nodules. Int J Mol Sci. (2023) 24. doi: 10.3390/ijms24076466 PMC10094849 37047439

[17] Beal EW Wei L Ethun CG Black SM Dillhoff M Salem A. Elevated NLR in gallbladder cancer and cholangiocarcinoma – making bad cancers even worse: results from the US Extrahepatic Biliary Malignancy Consortium. Hpb. (2016) 18:950–7. doi: 10.1016/j.hpb.2016.08.006 PMC5094484 27683047

[18] Gao Y Wu X Li Y Li Y Zhou Q Wang Q. The predictive value of MLR for radiation pneumonia during radiotherapy of thoracic tumor patients. Cancer Manage Res. (2020) 12:8695–701. doi: 10.2147/CMAR.S268964 PMC7518777 33061568

[19] Wang L Ruan M Yan H Lei B Sun X Chang C. Pretreatment serum neutrophil-to-lymphocyte and monocyte-to-lymphocyte ratios: Two tumor-related systemic inflammatory markers in patients with thymic epithelial tumors. Cytokine. (2020) 133. doi: 10.1016/j.cyto.2020.155149 32512341

[20] Ren W Zhang H Cheng L Zhang Y Yang C Nie L. Clinical significance of prognostic nutritional index (PNI)-monocyte-to-lymphocyte ratio (MLR)-platelet (PLT) score on postoperative outcomes in non-metastatic clear cell renal cell carcinoma. BMC Surg. (2023) 23. doi: 10.1186/s12893-023-02001-x PMC10170679 37165423

[21] Camp RL D-FM Rimm DL. X-tile: A new bio-informatics tool for biomarker assessment and outcome-based cut-point optimization. Clin Cancer Res. (2004) 24:7252–9. doi: 10.1158/1078-0432.CCR-04-0713 15534099

[22] Jakubzick C Gautier Emmanuel L Gibbings Sophie L Sojka Dorothy K Schlitzer A Johnson Theodore E. Minimal differentiation of classical monocytes as they survey steady-state tissues and transport antigen to lymph nodes. Immunity. (2013) 39:599–610. doi: 10.1016/j.immuni.2013.08.007 24012416 PMC3820017

[23] Zilionis R Engblom C Pfirschke C Savova V Zemmour D Saatcioglu HD. Single-cell transcriptomics of human and mouse lung cancers reveals conserved myeloid populations across individuals and species. Immunity. (2019) 50:1317–34.e10. doi: 10.1016/j.immuni.2019.03.009 30979687 PMC6620049

[24] Lavin Y Kobayashi S Leader A Amir E-a Elefant N Bigenwald C. Innate immune landscape in early lung adenocarcinoma by paired single-cell analyses. Cell. (2017) 169:750–65.e17. doi: 10.1016/j.cell.2017.04.014 28475900 PMC5737939

[25] Kennedy BC Showers CR Anderson DE Anderson L Canoll P Bruce JN. Tumor-associated macrophages in glioma: friend or foe? J Oncol. (2013) 2013:1–11. doi: 10.1155/2013/486912 PMC3664503 23737783

[26] Mariathasan S Turley SJ Nickles D Castiglioni A Yuen K Wang Y. TGFβ attenuates tumour response to PD-L1 blockade by contributing to exclusion of T cells. Nature. (2018) 554:544–8. doi: 10.1038/nature25501 PMC6028240 29443960

[27] Schmid D Park CG Hartl CA Subedi N Cartwright AN Puerto RB. T cell-targeting nanoparticles focus delivery of immunotherapy to improve antitumor immunity. Nat Commun. (2017) 8. doi: 10.1038/s41467-017-01830-8 PMC5700944 29170511

[28] Liu M O’Connor RS Trefely S Graham K Snyder NW Beatty GL. Metabolic rewiring of macrophages by CpG potentiates clearance of cancer cells and overcomes tumor-expressed CD47–mediated ‘don’t-eat-me’ signal. Nat Immunol. (2019) 20:265–75. doi: 10.1038/s41590-018-0292-y PMC6380920 30664738

[29] Kaneda Mm CP Nguyen AV Ralainirina N Hardamon CR Foubert P. Macrophage PI3Kγ Drives pancreatic ductal adenocarcinoma progression. Cancer Discovery. (2016) 8:870–85. 10.1158/2159-8290.CD-15-1346 PMC5091937 27179037

[30] Rachidi S Li H Wallace K Li Z Balch C Lautenschlaeger T. Preoperative platelet counts and postoperative outcomes in cancer surgery: a multicenter, retrospective cohort study. Platelets. (2020) 31:79–87. doi: 10.1080/09537104.2019.1573977 30744463

[31] Xu L Xu F Kong H Zhao M Ye Y Zhang Y. Effects of reduced platelet count on the prognosis for patients with non-small cell lung cancer treated with EGFR-TKI: a retrospective study. BMC Cancer. (2020) 20:1152. doi: 10.1186/s12885-020-07650-2 33243184 PMC7690006

[32] Zhou Q Huang F He Z Zuo MZ. Clinicopathological and prognostic significance of platelet count in patients with ovarian cancer. Climacteric. (2018) 21:60–8. doi: 10.1080/13697137.2017.1406911 29231068

