# Supplementary material for: Dynamic monocyte changes as prognostic indicators in operable gastric cancer: a retrospective cohort analysis
Source: Front Oncol. 2025 Feb 7;15:1514281. doi: 10.3389/fonc.2025.1514281 (PMC11842267; doi:10.3389/fonc.2025.1514281)
Supplement: Supplementary file 1 [file DataSheet1.docx]

**Dynamic Monocyte Changes as Prognostic Indicators in Operable Gastric Cancer: A Retrospective Cohort Analysis**

**Yiwei Jiang^1^, Xianwei Sun^2^,Chen yang^3^, Dandan Song^3^, Chongjun Zhou^3^, Xinxin Chen^1^, Chongquan Huang^4^ , Zhonglin Wang^3*^, Jiante Li^3*^**

^1^Department of Gastrointestinal Surgery, The 2nd Affiliated Hospital and Yuying Children’s Hospital of Wenzhou Medical University, Wenzhou 325000, Zhejiang Province, China

^2^Department of Gastrointestinal Surgery, The 1st Affiliated Hospital Wenzhou Medical University, Wenzhou 325000, Zhejiang Province, China

^3^Department of Anorectal Surgery, The 2nd Affiliated Hospital and Yuying Children’s Hospital of Wenzhou Medical University, Wenzhou 325000, Zhejiang Province, China

^4^Department of Radioimaging, Wenzhou Central Hospital, Wenzhou 325000, Zhejiang Province, China

**^*^**Address for correspondence: Jiante Li, The 2nd Affiliated Hospital and Yuying Children’s Hospital of Wenzhou Medical University, Wenzhou 325000, Zhejiang Province, China, E-mail: lijiante_wz@sina.cn.


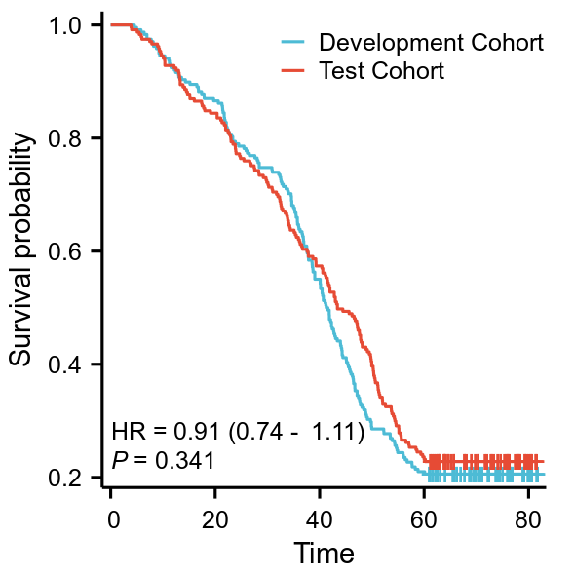


**Figure 1** The prognostic significance of two cohort.


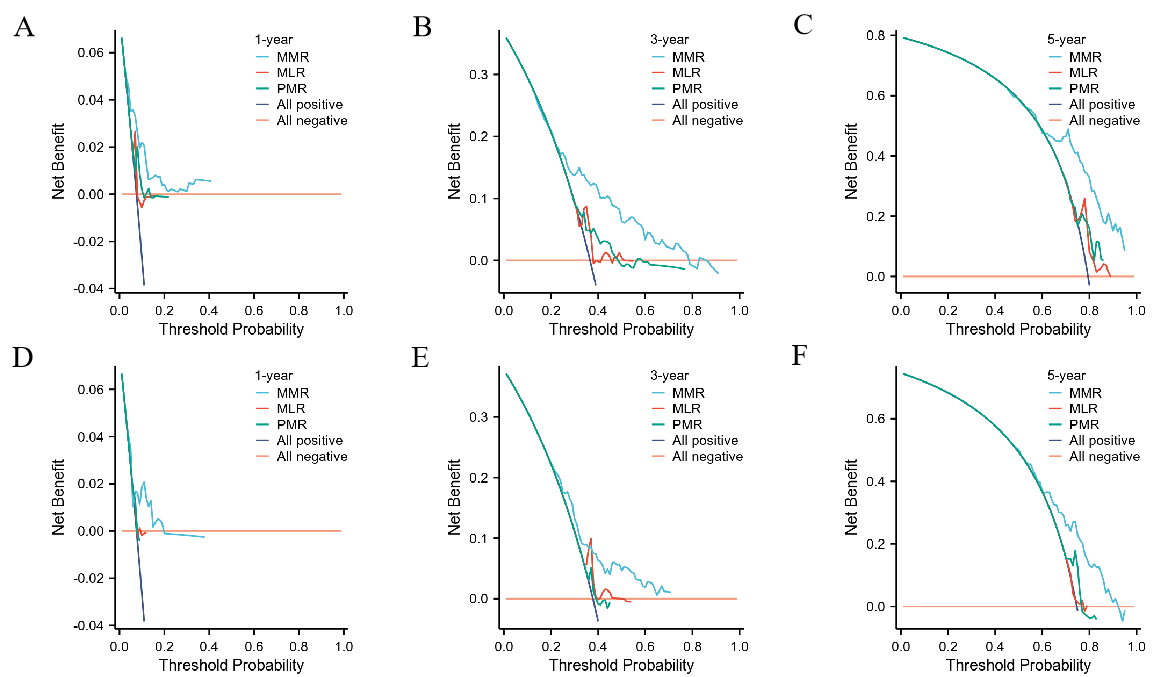


**Figure 2** Decision curve analysis of NLR, MMR, and MLR in Development cohort (A–C) and Teat cohort (D–F) in 1 year, 3 years, and 5 years
